# Screening for diagnostic targets in tuberculosis and study on its pathogenic mechanism based on mRNA sequencing technology and miRNA-mRNA-pathway regulatory network

**DOI:** 10.3389/fimmu.2023.1038647

**Published:** 2023-01-30

**Authors:** Yue Yang, Yujuan Fu, Siyu Sheng, Chunlei Ji, Xinyi Pu, Guangyu Xu

**Affiliations:** College of Pharmacy, Beihua University, Jilin, China

**Keywords:** mRNA sequencing, tuberculosis, diagnostic target, regulatory network, mechanism of action

## Abstract

**Purpose:**

Tuberculosis is common infectious diseases, characterized by infectivity, concealment and chronicity, and the early diagnosis is helpful to block the spread of tuberculosis and reduce the resistance of *Mycobacterium tuberculosis* to anti-tuberculosis drugs. At present, there are obvious limitations in the application of clinical detection methods used for the early diagnosis of tuberculosis. RNA sequencing (RNA-Seq) has become an economical and accurate gene sequencing method for quantifying transcripts and detecting unknown RNA species.

**Methods:**

A peripheral blood mRNA sequencing was used to screen the differentially expressed genes between healthy people and tuberculosis patients. A protein-protein interaction (PPI) network of differentially expressed genes was constructed through Search Tool for the Retrieval of Interacting Genes/Proteins (STRING) database. The potential diagnostic targets of tuberculosis were screened by the calculation of degree, betweenness and closeness in Cytoscape 3.9.1 software. Finally, the functional pathways and the molecular mechanism of tuberculosis were clarified in combination of the prediction results of key gene miRNAs, and by Gene Ontology (GO) enrichment analysis and the Kyoto Encyclopedia Genes and Genomes (KEGG) pathway annotation analysis.

**Results:**

556 Differential genes of tuberculosis were screened out by mRNA sequencing. Six key genes (AKT1, TP53, EGF, ARF1, CD274 and PRKCZ) were screened as the potential diagnostic targets for tuberculosis by analyzing the PPI regulatory network and using three algorithms. Three pathways related to the pathogenesis of tuberculosis were identified by KEGG pathway analysis, and two key miRNAs (has-miR-150-5p and has-miR-25-3p) that might participate in the pathogenesis of tuberculosis were screened out by constructing a miRNA-mRNA pathway regulatory network.

**Conclusion:**

Six key genes and two important miRNAs that could regulate them were screened out by mRNA sequencing. The 6 key genes and 2 important miRNAs may participate in the pathogenesis of infection and invasion of *Mycobacterium tuberculosis* through herpes simplex virus 1 infection, endocytosis and B cell receptor signaling pathways.

## 1 Introduction

Tuberculosis (TB) is a chronic infectious disease caused by *Mycobacterium tuberculosis* (1). According to the World Health Organization (WHO), there were 5.8 million new cases and 1.5 million TB deaths in 2021 (2), and the long-term latency and drug resistance of *Mycobacterium tuberculosis* have not been solved yet (3). It is necessary to take adequate prevention and treatment measures to reduce the pain and economic loss of patients with pulmonary tuberculosis. The traditional single gene screening for targets of anti-tuberculosis drugs can not meet the needs of clinical medicine (4), and the existing early diagnosis technology of tuberculosis can not keep up with the development of the times (5, 6). Screening new drug targets and diagnostic targets for tuberculosis by mRNA sequencing technology may provide a new breakthrough for our existing research.

RNA sequencing (RNA-Seq) has become an economical and accurate gene sequencing method for quantifying transcripts and detecting unknown RNA species (7). So far, it has been proven that the application of RNA sequencing can be used to predict relevant clinical outcomes, and has been increasingly used in the diagnosis of diseases to achieve a personalized treatment, bringing a new insight into the transcriptional biology at the same time (8, 9). In this study, differentially expressed genes of tuberculosis patients and healthy people were screened by peripheral blood mRNA sequencing technology, potential diagnostic targets of tuberculosis were screened by constructing PPI regulatory network and establishing various algorithms (10), and miRNAs regulated by them were predicted and miRNA-mRNA regulatory network was constructed to clarify the possible mechanism of tuberculosis, which was attempted to lay a foundation for the early clinical diagnosis of tuberculosis and the development of new anti-tuberculosis drugs.

## 2 Materials and methods

### 2.1 Data source and grouping

A total of 20 blood samples was collected, including those from 10 patients with pulmonary tuberculosis (**Supplementary Material table 1**) and 10 healthy controls (**Table 1**). The 20 volunteers were all over 20 years old, without a history of previous malignancies, and all of them signed the written informed consent approved by the Ethics Committee of the Affiliated Hospital of Beihua University abd the committee also approved this study.

**Table 1:** Basic information for 10 healthy individuals.

Health groups	Sex	Tuberculin test
1	male	negative
2	female	negative
3	female	negative
4	male	negative
5	female	negative
6	female	negative
7	female	negative
8	female	negative
9	male	negative
10	female	negative

### 2.2 mRNA sequencing

The whole blood samples of 20 volunteers were obtained by antecubital venipuncture. Total RNA was extracted from whole blood with the PAXgene Blood RNA Kit. The total RNA of whole blood samples was purified using Agilent Bioanalyzer 2100 (Agilent Technologies, USA) for quality control. The RNA with RIN ≥ 7.0 (RNA Integrity Number) was used to ensure the construction of the downstream high-quality total RNA SEQ library. The sequencing conditions were: RNA sample concentration ≥ 100 ng/µL, total amount > 2 μg, OD260/280 values between 1.8 and 2.2, and OD260/230 ≥ 2.0. Finally, the library was sequenced using 2 × 150 bp double ended sequencing strategy on an Illumina Hiseq 2500 high-throughput sequencing platform.

### 2.3 Construction of PPI regulatory network

STING database (http://www.string-db.org/) is a protein interaction database that can be used to search for known and predicted protein-protein interactions (11). In this study, the differentially expressed genes of the screened tuberculosis patients and healthy controls were entered into the STING database and the study species were selected as human (“Homo sapiens”) and the free nodes were removed to obtain the differential gene PPI network. The PPI network was visualized using the CytoNCA plug-in for network centrality analysis in Cytoscape 3.9.1 software (12).

### 2.4 Screening of potential key genes

The degree betweenness and closeness of differential genes in the PPI network were calculated by three algorithms: degree centrality (DC) that judges the importance or influence of nodes in the network, betweenness centrality (BC) that represents the degree of interaction between nodes and closeness centrality (CC) that measures the average shortest distance between nodes and other points in their connected components (13, 14). The calculation formulas are as follows:

#### 2.4.1 Degree centrality:


$$\text{D}{\text{C}}_{\text{i}}=\frac{{\text{k}}_{\text{i}}}{\text{N}-1}$$

#### 2.4.2 Betweeness centrality:


$${\text{BC}}_{\text{i}}=\sum_{\text{s}\neq \text{i}\neq \text{t}}\frac{{\text{n}}_{\text{st}}^{\text{i}}}{{\text{g}}_{\text{st}}}$$

#### 2.4.3 Closeness centrality:


$$d_{i}=\frac{1}{N-1}\sum_{j=1}^{N}d_{ij}\;\;\;\;\;\;\;\;\;\;\;\;\;CC_{i}=\frac{1}{d_{i}}$$

### 2.5 GO functional clustering analysis and KEGG pathway enrichment analysis

The screened differentially expressed genes were submitted to the Database for Annotation, Visualization and Integrated Discovery (DAVID) (https://david.ncifcrf.gov/tools.jsp) (15), and Homo sapiens was taken as the research object. Gene Ontology (GO) (16) clustering analysis and Kyoto Encyclopedia of genes and genomes (KEGG) (17) pathway analysis were performed on the screened differential genes using the functional annotation module in DAVID.

### 2.6 Statistical analysis

GraphPadPrism 8 software was used for mapping and t-test. P< 0.05 was considered to be statistically significant.

## 3 Results

### 3.1 mRNA sequencing and data analysis

**Figure 1:**
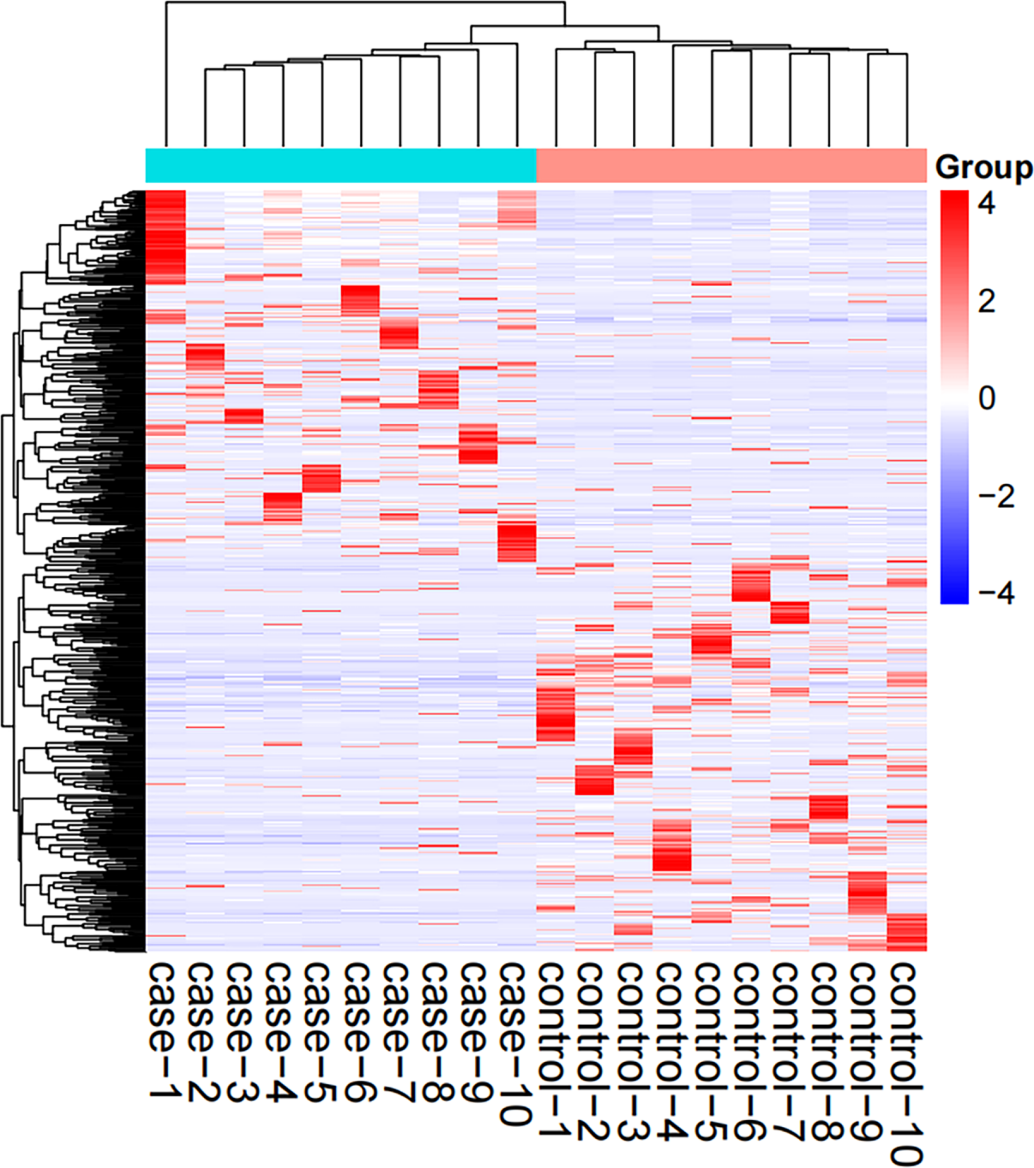
Hotspot map of mRNA sequencing results. Case: tuberculosis group; Control: healthy control group; Red: up-regulated differential genes; Blue: down-regulated differential genes.

**Figure 2:**
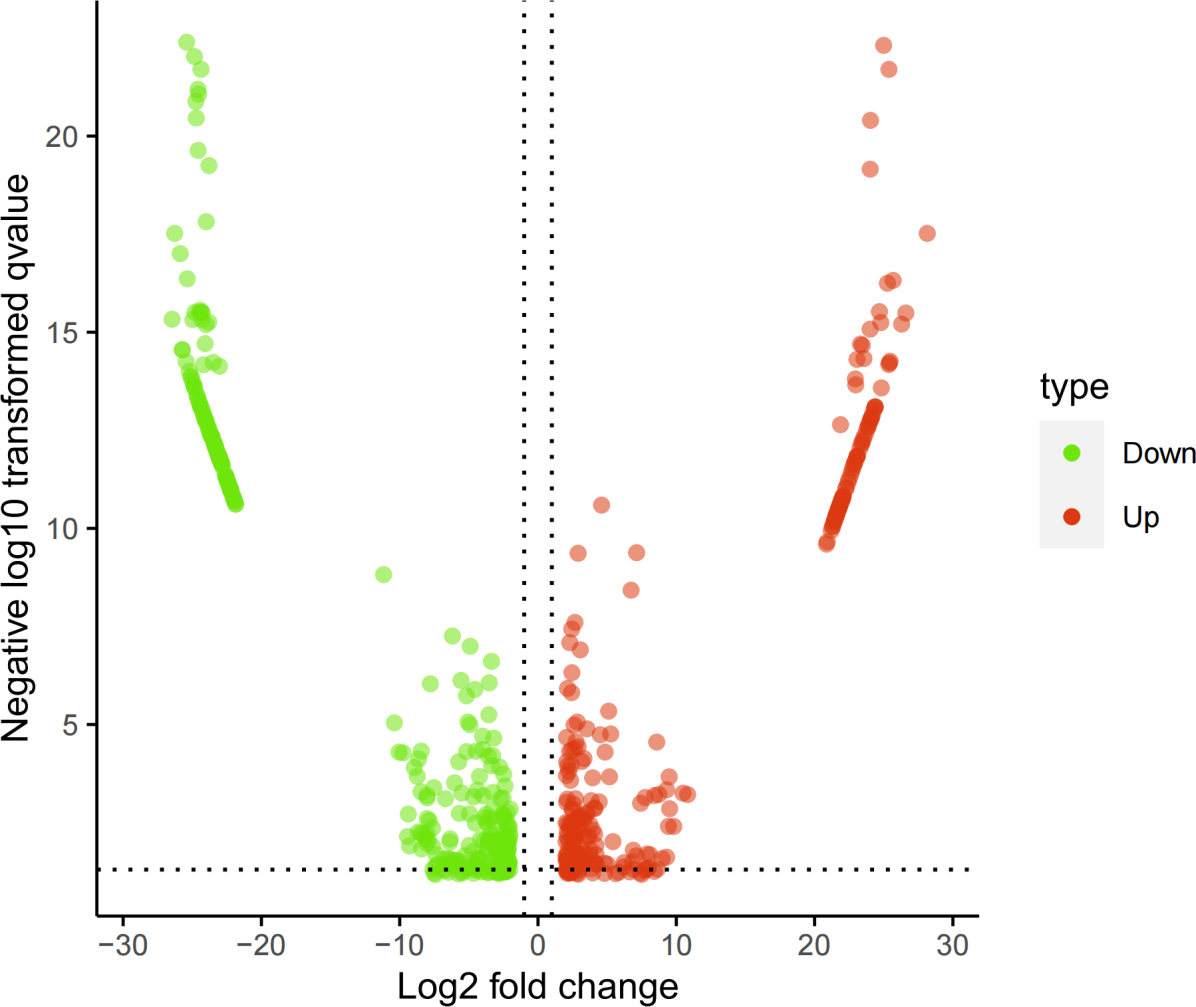
Volcano map of mRNA sequencing results. Red: up-regulated differential genes; Green: down-regulated differential genes.

The screened 556 differentially expressed genes (**Supplementary Material table 2**) between healthy people and tuberculosis patients were screened by peripheral blood mRNA sequencing with P value< 0.005 and |log FC| ≥ 2 as the setting conditions, of which 256 genes were up-regulated and 300 genes were down-regulated (**Figures 1**, **2**).

### 3.2 Differential gene PPI network

**Figure 3:**
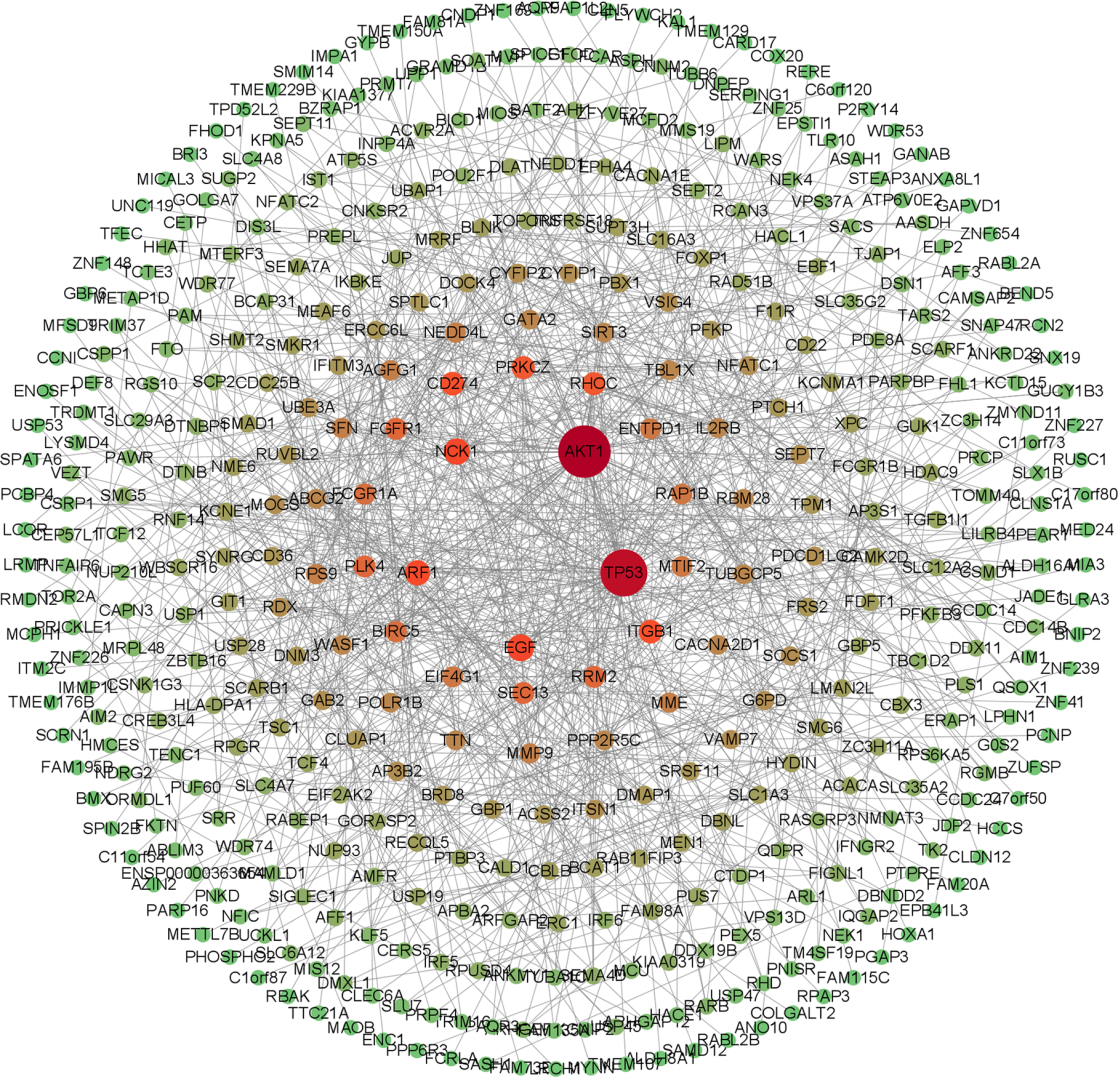
PPI regulatory network diagram. The network color is the progressive distribution from green to red, and the node size is the progressive arrangement from small to large. The redder the node and the larger, the greeter the degree of the node, and the greener the node, the smaller the degree of the node.

The screened 556 differentially expressed genes were entered into the STRING database, and free genes were removed through hide disconnected nodes in the network to further obtain 430 important genes. The PPI regulatory network was visualized using Cytoscape 3.9.1 software (**Figure 3**), and there were 430 nodes and 909 edges in the PPI regulatory network.

### 3.3 Screening of potential key genes of tuberculosis

**Figure 4:**
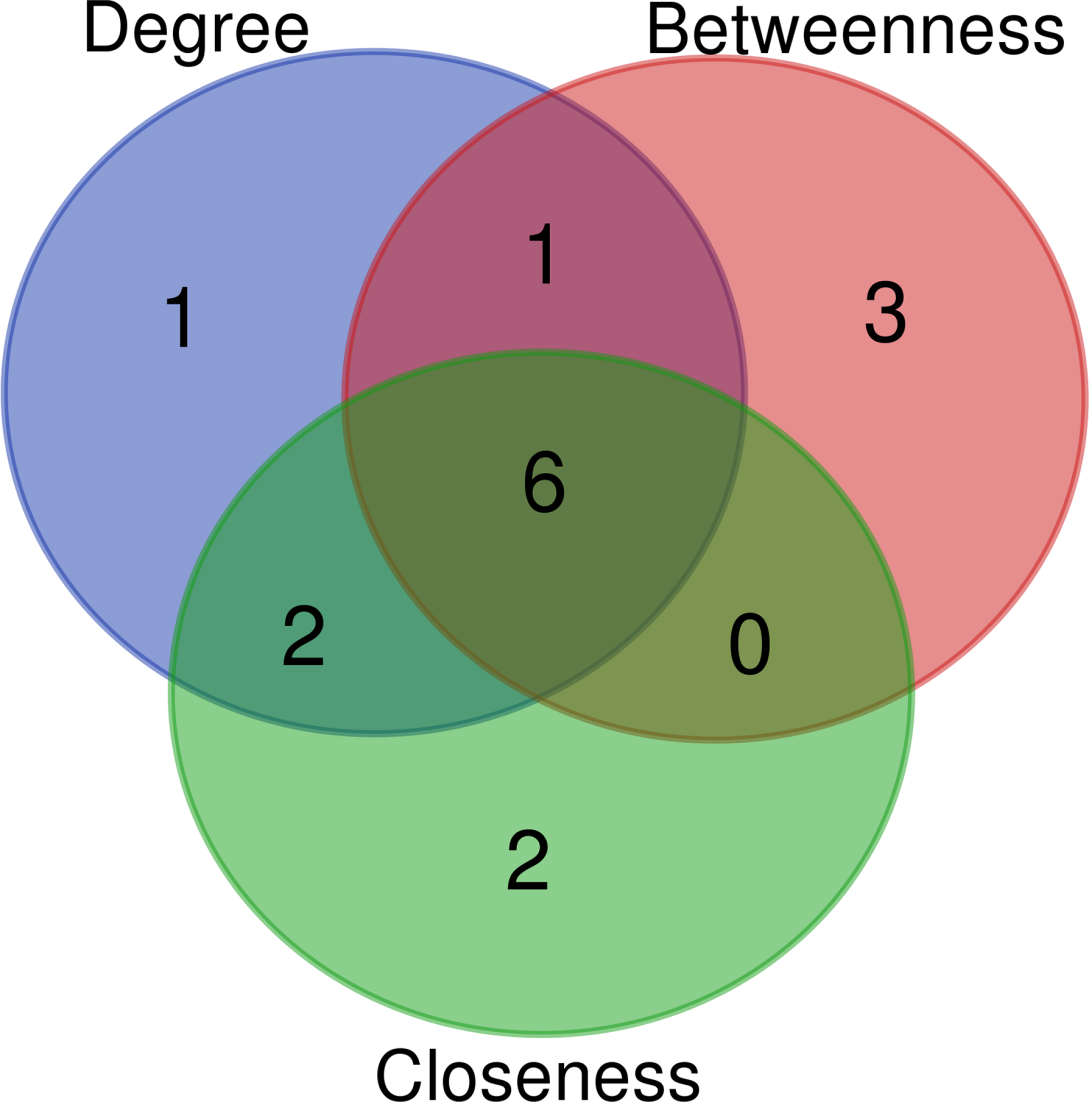
Wayne diagram of intersection of top ten key nodes by DC, BC and CC algorithms.Blue: top ten key nodes by DC algorithm; Red: top ten key nodes by BC algorithm; Green: top ten key nodes by CC algorithm.

The key genes from 430 differential genes in the PPI regulatory network were screened out by three different algorithms, DC, BC and CC. The top ten genes screened by each algorithm were set as the core genes (**Table 2**) and the intersection screening of them was performed by using Venn diagram (**Figure 4**), and finally, six key genes (AKT1, TP53, EGF, ARF1, CD274 and PRKCZ) were obtained. These six key genes were considered more likely to be the potential diagnostic targets of tuberculosis (**Table 3**).

**Table 2:** Top 10 differential genes ranked by degree, betweenness and closeness values.

key nodes	Degrree	key nodes	Betweeness	key nodes	Closeness
AKT1	69	TP53	60851.12161	AKT1	0.398698885
TP53	59	AKT1	56959.60582	TP53	0.395027624
EGF	22	ARF1	15621.89434	EGF	0.34047619
NCK1	21	EGF	10192.68202	ARF1	0.33411215
ARF1	19	NCK1	10033.49694	PRKCZ	0.332300542
ITGB1	16	RAP1B	9468.955752	RHOC	0.331786543
CD274	16	PRKCZ	8071.668674	ITGB1	0.331274131
PRKCZ	15	EIF4G1	7495.967994	PPP2R5C	0.33
RHOC	15	CD274	7490.923055	CD274	0.328735632
FGFR1	13	RRM2	7213.222312	CYFIP2	0.324263039

**Table 3:** Intersection data results of top ten key nodes by DC, BC and CC algorithms.

Name	Total	Elements
Betweenness and Closeness and Degree	6	AKT1、TP53、EGF、ARF1、CD274、PRKCZ
Betweenness and Degree	1	NCK1
Closeness and Degree	2	ITGB1、RHOC
Degree	1	SEC13
Betweenness	3	RAP1B、RRM2、EIF4G1
Closeness	2	PPP2R5C、CYFIP2

### 3.4 GO clustering analysis and KEGG gene pathway enrichment analysis

#### 3.4.1 GO cluster pathway analysis

**Figure 5:**
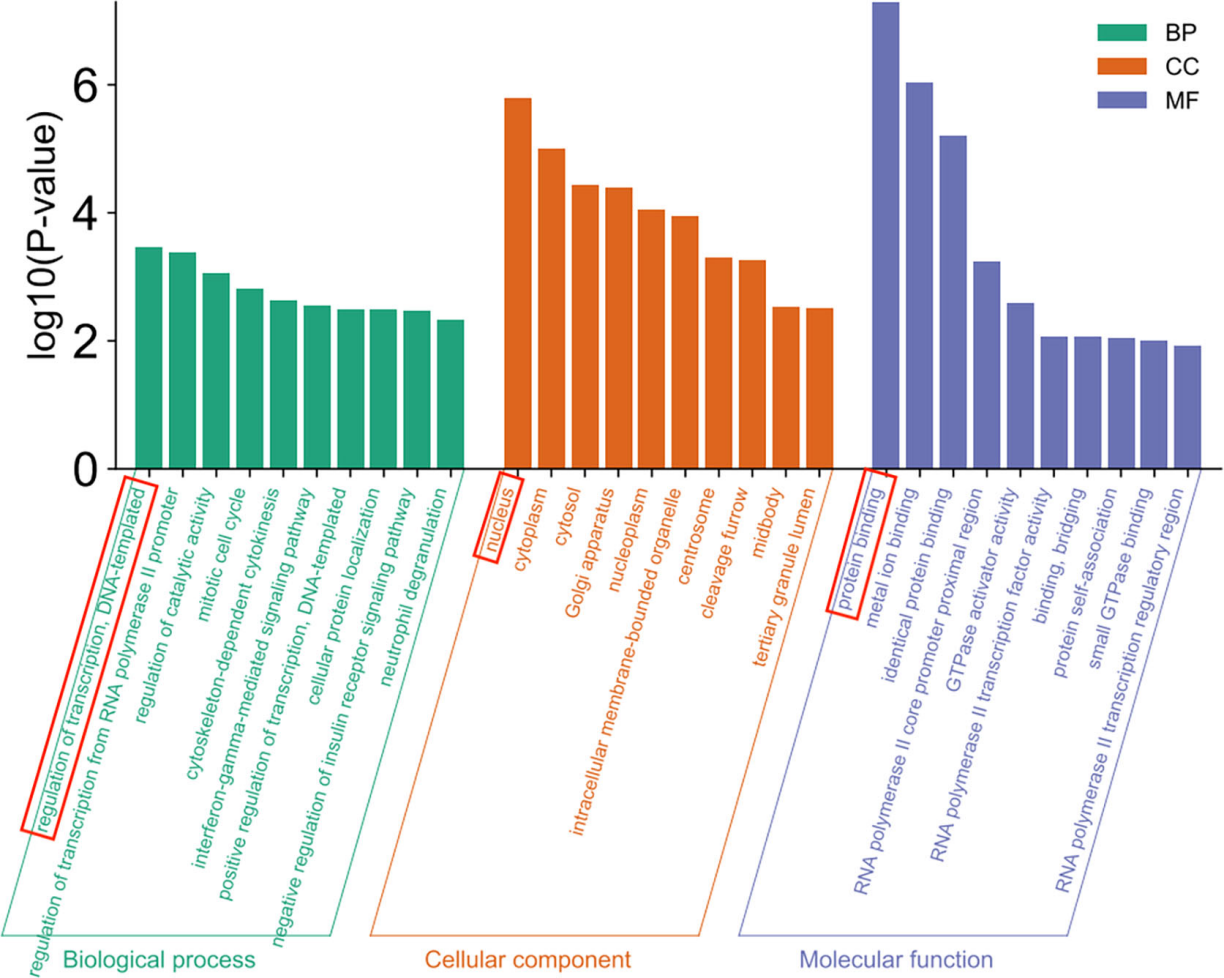
GO enrichment analysis.

GO clustering analysis including biological process (BP), cellular component (CC) and molecular function (MF) was performed on 556 differential genes using DAVID database, and a total of 166 pathways were found. Among them, 79 pathways were related to BP, and the pathways with the highest significance of P-value were regulation of transcription and DNA-templated, 50 pathways were correlated with CC and the pathway with the highest P-value was the nucleus, and 37 pathways were associated with MF and the pathway with the highest significance of P-value was protein binding (**Figure 5**).

### 3.5 KEGG enrichment pathway analysis

**Figure 6:**
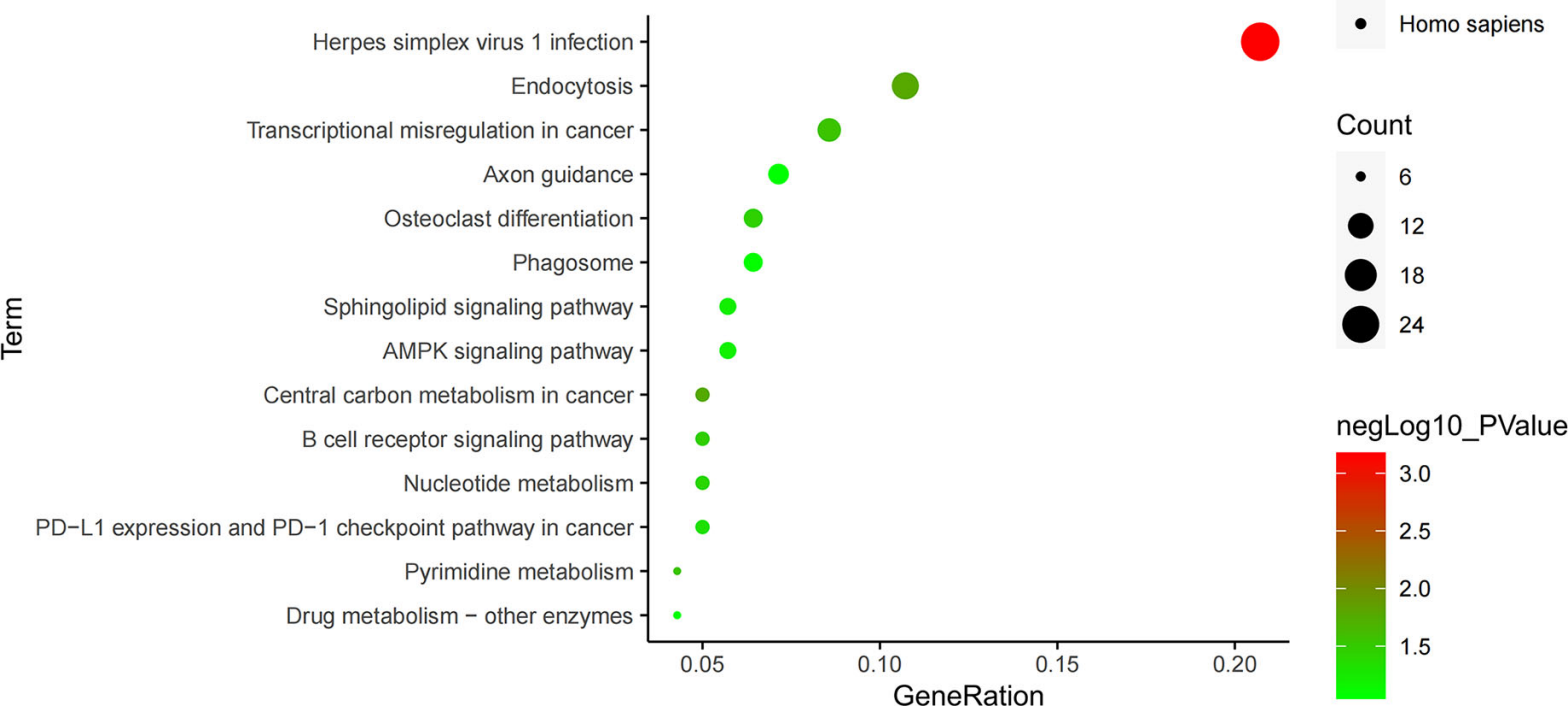
KEGG pathway enrichment analysis.

The enrichment pathways of 556 differential genes were also analyzed through KEGG database, and 14 pathways were found, which were arranged from small to large according to their significance (P value) (**Figure 6**). Among them, the pathway with the highest significance was the herpes simplex virus 1 infection pathway, closely related to the infection mechanism of tuberculosis, and the endocytosis pathway was the second most significant pathway, closely related to the invasive mechanism of tuberculosis. There were three signaling pathways in these pathways, of which B cell receptor signaling pathway was an immune-related pathway with a higher significance, while AMPK signaling pathway and sphingolipid signaling pathway had no significance (**Table 4**).

**Table 4:** KEGG enrichment pathways with significance.

Pathway	Gene number	PValue
Herpes simplex virus 1 infection	29	0.000664519
Endocytosis	15	0.016634411
Central carbon metabolism in cancer	7	0.016965457
Transcriptional misregulation in cancer	12	0.027251953
Pyrimidine metabolism	6	0.027791341
B cell receptor signaling pathway	7	0.033930065
Osteoclast differentiation	9	0.035590242
Nucleotide metabolism	7	0.039437744
PD-L1 expression and PD-1 checkpoint pathway in cancer	7	0.047620204

### 3.6 Construction of miRNA-mRNA-pathway regulatory network

To further elucidate the regulatory mechanism of key genes, miRNAs associated with six key genes (AKT1, TP53, EGF, ARF1, CD274 and PRKCZ) were searched through mirWalk database (http://miRWalk.umm.uni-heidelberg.de/). MiRNAs that could be annotated on Targetscan database, miRDB database and mirTarBASE database at the same time were directly screened by using mirWalk database as our research objects. It was found that two key miRNAs (has-mir-150-5p and has-mir-25-3p) were closely related to these six genes (**Supplementary Material table 3**).

**Figure 7:**
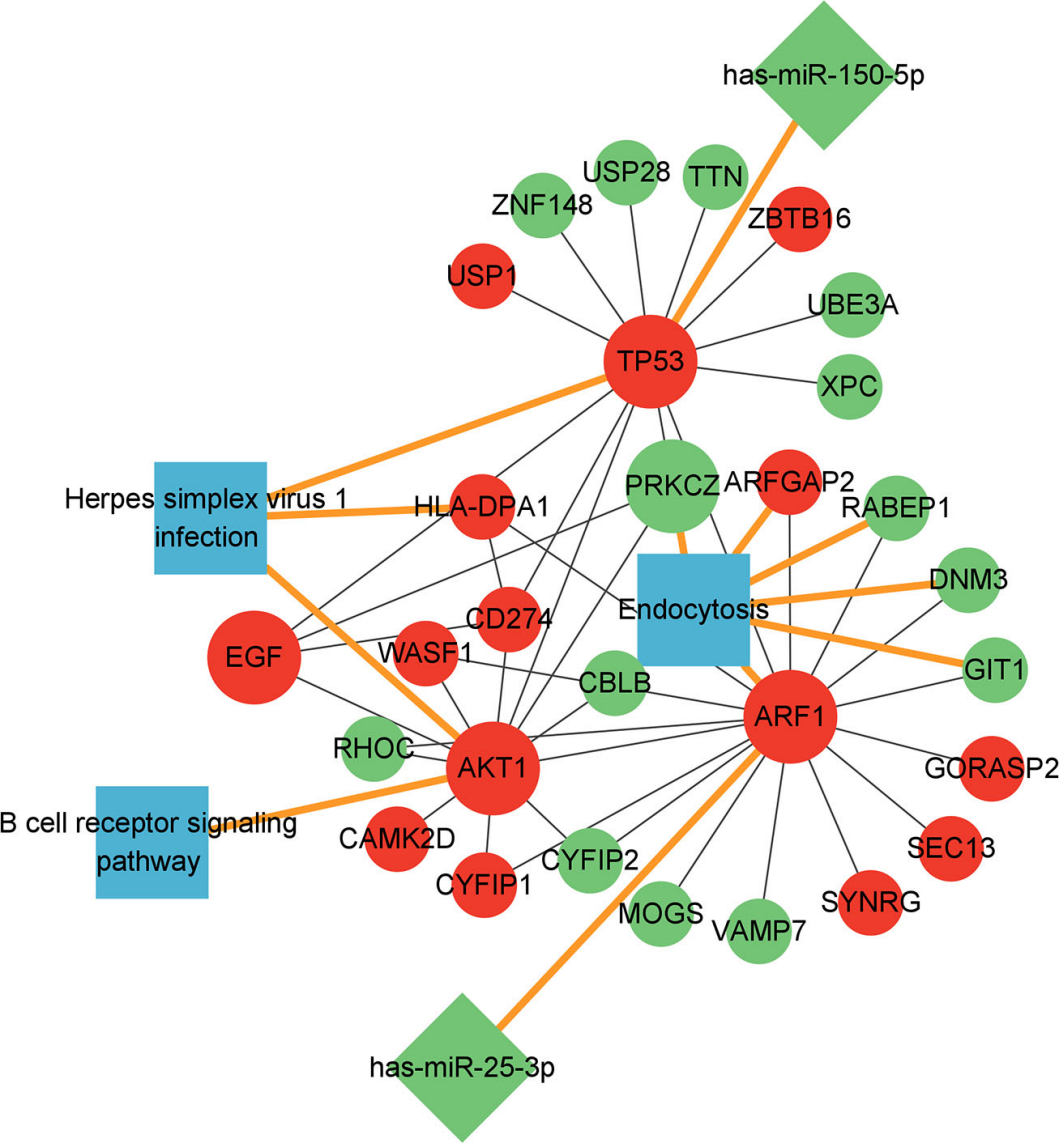
miRNA-mRNA-pathway regulatory network diagram.Red: up-regulated expression genes; Green: down-regulated expression genes; Blue: related pathways; Round: mRNA, the larger circle represents the 6 key mRNAs, the diamond represents the miRNA, and the square represents the related pathway. mRNA-miRNA and mRNA-pathway was emphasized with yellow lines.

The six key genes and two corresponding miRNAs, other mRNAs in the PPI regulatory network related to these six key genes and the three pathways where these six genes were located were used together to construct the miRNA-mRNA-pathway regulatory network (**Figure 7**).

It was found in the miRNA-mRNA-pathway regulatory network that the two miRNAs has-miR-150-5p and has-miR-25-3p could regulate gene TP53 and ARF1, respectively, and the expression of gene TP53 and ARF1 was up-regulated because the expression of these two miRNAs was inhibited. The up-regulated expression of gene TP53 and ARF1 could activate the expression of AKT1, EGF and CD274 and activate the pathway herpes simplex virus 1 infection and B cell receptor signaling pathway. At the same time, the up-regulated expression of TP53 and ARF1 suppressed the expression of PRKCZ and the endocytosis pathway.

## 4 Discussion

Pulmonary tuberculosis is an infectious disease of respiratory system caused by *Mycobacterium tuberculosis*, and the main cause of infectious death in the world, caused 1.5 million deaths in 2021 (2). The current clinical diagnosis and treatment technology of tuberculosis have not kept up with the development of the times, so it is urgent for us to need innovative diagnosis methods that can diagnose it rapidly at its early stage and new effective anti-tuberculosis drugs that can shorten the treatment time and improve the prevention and treatment effect (6). In this study, the potential diagnostic targets of tuberculosis were screened out through mRNA sequencing technology, which may provide a theoretical basis for the early diagnosis of tuberculosis in clinics and the development of specific new drugs used for the prevention and treatment of tuberculosis.

First, 556 differentially expressed genes of tuberculosis were screened out, then the PPI regulatory network was constructed and the key nodes of the PPI regulatory network through were screened out by the combination of three algorithms (DC, BC and CC), and finally we found six key genes (AKT1, TP53, EGF, ARF1, CD274 and PRKCZ).

Among the six key genes, the protein expressed by AKT1 gene is the key enzyme in controlling the intracellular growth in tuberculosis, and some studies have pointed out that it may be a well candidate for genetic markers for assessing tuberculosis risks (18). TP53 gene can regulate the immune function of human body to evade attacks, eventually leading to the existing of *Mycobacterium tuberculosis* in a non-proliferative state in the host through its impact on the host immune system to cause the delay of the disease course and the repeated and persistent infection (19). EGF gene is the growth factor of pathogenic mycobacteria in granuloma tissue and macrophages, and may improve the growth rate of intracellular and extracellular mycobacteria at the infection site (20). Moreover, it has been demonstrated that EGF combined with the other four tuberculosis specific biomarkers can accurately predict 90.9-100% of active pulmonary tuberculosis (21). ARF1 gene can affect the rearrangement of actin cytoskeleton in epithelial cells, and the initial activation of ARF1 can promote the invasion of mycobacteria (22). CD274, also known as programmed cell death ligand 1 (PD-L1), is associated with the suppression of the immune system (23). It has been found that *Mycobacterium tuberculosis* can promote the expansion of regulatory T cells by inducing CD274 gene in dendritic cells (24). In addition, it has been shown that 10 biomarkers, including CD274 and EGF, can be used for the diagnosis of tuberculosis, the differential diagnosis of latent tuberculosis infection/active tuberculosis, and the risk of progression to active tuberculosis (25). PRKCZ is a member of the PKC family, and the gene level of the PKC family can be used as a predictor of response to immunotherapy (26). The above analysis indicates that these six genes (AKT1, TP53, EGF, ARF1, CD274 and PKCZ) are all related to the immune system of the host and involved in the pathogenesis of tuberculosis, EGF and CD274 have been used as biomarkers for the diagnosis of tuberculosis (21, 25). Therefore, we suggest that EGF and CD274 are potential drug targets and diagnostic targets for TB, and AKT1, TP53, ARF1 and PRKCZ may be new potential drug targets and diagnostic targets for TB.

The GO enrichment analysis showed that the enrichment of regulation of transcription and DNA-templated was the most significant in biological process (BP), which may be because the transcription process is the first step of protein biosynthesis, and DNA-templated is one of the indispensable conditions in the transcription process; the enrichment of the nucleus was the most significant in the cellular component (CC), and it was found in the study that the proteins contained in *Mycobacterium tuberculosis* can ectopic to the host nucleus to inhibit the production of nitric oxide (27), which may be because the nucleus is the control center of cells genetic and metabolic activities; the enrichment of protein binding was the most significant in molecular function (MF). The results of GO enrichment analysis indicate that *Mycobacterium tuberculosis* may affect the protein binding function through the first step of protein biosynthesis, and then cause the effector protein of *Mycobacterium tuberculosis* to enter the host nucleus to regulate the host gene expression (28).

In the KEGG pathway analysis of differential genes, 14 pathways were screened out, of which the most significant pathway was the herpes simplex virus 1 infection pathway, the herpes virus infection pathway. Some studies have found that patients with pulmonary tuberculosis have a higher active infection of herpes simplex virus (29), so this pathway may also be related to the pathogenesis of tuberculosis. Endocytosis pathway was the second most significant pathway, and *Mycobacterium tuberculosis* often invades cells through endocytosis, so this pathway is closely related to the invasion mechanism of *Mycobacterium tuberculosis* (30). B cell receptor signaling pathway, an important immune pathway, can regulate the survival, proliferation and differentiation of B lymphocytes (31, 32). In addition, we also found that among the six key genes previously screened, TP53 and AKT1 genes were in the herpes simplex virus 1 infection pathway, genes ARF1 and PRKCZ were in the endocytosis pathway, and gene AKT1 was in the B cell receptor signaling pathway, indicating that these four genes all play important regulatory roles in the pathway. Therefore, it was speculated that genes AKT1, TP53, EGF, ARF1, CD274 and PRKCZ may cause the infection and invasion of *Mycobacterium tuberculosis* through herpes simplex virus 1 infection, endocytosis and B cell receptor signaling pathways.

A miRNA-mRNA-pathway regulatory network was constructed by using the six key genes (AKT1, TP53, EGF, ARF1, CD274 and PRKCZ), two miRNAs (has-mir-150-5p and has-mir-25-3p) and three pathways (herpes simplex virus 1 infection, endocytosis and B CLL receptor signaling pathway) together. The analysis of the miRNA-mRNA-pathway regulatory network showed that two miRNAs could regulate genes TP53 and ARF1, respectively. MiR-150, a developmental regulator of several immune cell types, can regulate the differentiation of B cells/T cells/natural killer cells (33). In addition, by reviewing the literature, we found that previous studies have proved that has-miR-25-3p is down-regulated in tuberculosis patients by RT-PCR experiments (34).

Furthermore, we also found that gene TP53 was in the herpes simplex virus 1 infection pathway, and gene AKT1 could play a regulatory role not only in the herpes simplex virus 1 infection pathway but also in the B cell receptor signaling pathway, suggesting genes TP53 and AKT1 may play a key role in regulating the immunity and the intracellular growth of *Mycobacteria tuberculosis*. In addition to the key genes TP53 and AKT1 in the herpes simplex virus 1 infection pathway, we found that the up-regulated target gene HLA-DPA1 corresponding to both EGF and TP53 could also play a regulatory role in the herpes simplex virus 1 infection pathway. So it is speculated that the up-regulated genes TP53, AKT1 and HLA-DPA1 may activate the herpes simplex virus 1 infection pathway in the body, leading to the occurrence of tuberculosis. Besides genes ARF1 and PRKCZ, it was found in the endocytosis pathway that the up-regulated gene ARFGAP2 corresponding to ARF1 and the down-regulated genes RABEP1, DNM3 and GIT1 were also in the endocytosis pathway, so it is speculated that endocytosis’s cell surface receptor internalization and signal regulation functions may be disordered by the down-regulated genes PRKCZ, RABEP1, DNM3 and GIT1, and The up-regulated expression of ARF1 and ARFGAP2 may cause the endocytosis mechanism of host cells to be hijacked by the pathogen, leading to the invasion of *Mycobacterium tuberculosis* into the body.

Therefore, we speculate that in tuberculosis patients, the expression of genes AKT1 and TP53 is up-regulated, and the herpes simplex virus 1 infection pathway is activated at the same time, so that *Mycobacterium tuberculosis* is rapidly infected, and in addition, gene AKT1 may activate the B cell receptor signaling pathway, that is, activating the human immune system, and at the same time, gene PRKCZ may inhibit the endocytosis pathway to inhibit the endocytosis, so that the infection of *Mycobacterium tuberculosis* cannot be suppressed by endocytosis, thereby accelerating the pathogenesis of tuberculosis.

## 5 Conclusion

Six key genes and 2 miRNAs that regulate them were screened out by mRNA sequencing, and PPI regulatory network construction and analysis. They may regulate the pathogenesis of infection and invasion of *Mycobacterium tuberculosis* through the herpes simplex virus 1 infection, endocytosis and B cell receptor signaling pathway, which may lay a foundation for the early clinical diagnosis and research of tuberculosis and the development of new anti-tuberculosis drugs.

## Data availability statement

The datasets presented in this study can be found in online repositories. The names of the repository/repositories and accession number(s) can be found below: https://www.ncbi.nlm.nih.gov/bioproject/PRJNA876021. SRA data: PRJNA876021.

## Ethics statement

The studies involving human participants were reviewed and approved by medical Ethics Committee of Beihua University Affiliated Hospital. The patients/participants provided their written informed consent to participate in this study.

## Author contributions

YY wrote the manuscript and derived the datasets, YF and SS revised the manuscript, CJ and XP performed data analysis. GX designed the study. All authors contributed to the article and approved the submitted version.
